# Practical guidelines addressing ethical issues pertaining to the curation of human locus-specific variation databases (LSDBs)

**DOI:** 10.1002/humu.21339

**Published:** 2010-08-03

**Authors:** Sue Povey, Aida I Al Aqeel, Anne Cambon-Thomsen, Raymond Dalgleish, Johan T den Dunnen, Helen V Firth, Marc S Greenblatt, Carol Isaacson Barash, Michael Parker, George P Patrinos, Judith Savige, Maria-Jesus Sobrido, Ingrid Winship, Richard GH Cotton

**Affiliations:** 1 Department of Genetics, Evolution and Environment, University College LondonLondon, United Kingdom; 2 Department of Paediatrics, Riyadh Military Hospital, Department of Genetics and Stem Cell Therapy Program, King Faisal Specialist Hospital and Research CentreKingdom of Saudi Arabia; 3 UMR Inserm, Université Paul Sabatier—Toulouse IIIToulouse, France; 4 Department of Genetics, University of LeicesterLeicester, United Kingdom; 5 Human and Clinical Genetics, Leiden University Medical CentreLeiden, The Netherlands; 6 Department of Medical Genetics, Addenbrooke's HospitalCambridge, United Kingdom; 7 University of Vermont College of MedicineBurlington, Vermont; 8 Genetics, Ethics & Policy ConsultingBoston, Massachusetts; 9 Ethox Centre, Department of Public Health and Primary Health Care, University of Oxford, HeadingtonOxford, United Kingdom; 10 University of Patras, School of Health Sciences, Department of PharmacyPatras, Greece; 11 Department of Medicine, University of MelbourneEpping, Australia; 12 Fundacion Publica Galega de Medicina XenomicaSantiago de Compostela. Spain; 13 Center for Network Biomedical Research on Rare Diseases (CIBERER), Institute of Health Carlos IIIMadrid, Spain; 14 Department of Clinical Genetics, University of Melbourne, Royal Melbourne HospitalMelbourne, Victoria, Australia; 15 Faculty of Medicine, Dentistry and Health Sciences, The University of Melbourne, Royal Melbourne HospitalParkville, Australia; 16 Genomic Disorder Research Centre, Howard Florey InstituteCarlton South, Victoria, Australia

**Keywords:** phenotype, LSDB, data-sharing, ethics, curators, guidelines, diagnostic test, consent

## Abstract

More than 1,000 Web-based locus-specific variation databases (LSDBs) are listed on the Website of the Human Genetic Variation Society (HGVS). These individual efforts, which often relate phenotype to genotype, are a valuable source of information for clinicians, patients, and their families, as well as for basic research. The initiators of the Human Variome Project recently recognized that having access to some of the immense resources of unpublished information already present in diagnostic laboratories would provide critical data to help manage genetic disorders. However, there are significant ethical issues involved in sharing these data worldwide. An international working group presents second-generation guidelines addressing ethical issues relating to the curation of human LSDBs that provide information via a Web-based interface. It is intended that these should help current and future curators and may also inform the future decisions of ethics committees and legislators. These guidelines have been reviewed by the Ethics Committee of the Human Genome Organization (HUGO). Hum Mutat 31:–6, 2010. © 2010 Wiley-Liss, Inc.

## Introduction

This document is designed to assist a curator who intends to provide access to the information contained in a human Locus Specific variation Database (LSDB). For this purpose, an LSDB is defined as a listing of known sequence variants in a specific human gene together with some assessment of the effects of these variants on the phenotype. It may also highlight the frequency of both common and rare variants (e.g., single nucleotide polymorphisms) prevalent in particular populations groups. Although ethical issues arise in a database of any format, currently the access is nearly always provided via a Web interface, usually available to everyone but occasionally restricted to selected professional groups. An example of a well-known LSDB that can be accessed by anyone is that describing the mutations in the gene *DMD*, deficient in Duchenne Muscular Dystrophy, curated by one of the authors (J.dD.) in Leiden (http://www.dmd.nl/nmdb2/home.php). This format is available in open-source software and is now used in many other LSDBs. The need for these guidelines has been highlighted by the recognition by initiators of the Human Variome Project (HVP) of the immense unpublished and inaccessible resource of information existing in diagnostic laboratories and the significant clinical need to have access to this information [Cotton et al., 2007; Kaput et al., 2009].

These guidelines are largely an expansion in detail of the first generation guidelines proposed by Cotton and coauthors in 2005 [Cotton et al., 2005], which were rooted in the principles described by Knoppers and Laberge in 2000 [Knoppers and Laberge, 2000]. They were discussed and modified as a result of the international HVP planning meeting in Spain May 2008 attended by participants from a wide range of developed and emerging countries [Kaput et al., 2009]. Details of this can be found in the published meeting report and its supplementary information [Kaput et al., 2009]. The content and order of headings in the current version differs slightly from that of Cotton et al. [2005] and is shown in Table 1.

**Table 1:** 12 Major Points to Consider Pertaining to Ethical Issues Arising in the Curation of Human Locus-Specific Variation Databases (LSDBs)

1. Clarify the main purpose of the particular database
2. Define database policy with respect to sources of data
3. Take specific communities/cultures into account
4. Take vulnerable persons into account
5. Create an ethics oversight committee
6. Remove identifying information before submission to the database
7. Add further protection of confidentiality if needed
8. Allow no further disclosure without consent
9. Make provision for removal of data from the database
10. Be cautious in response to requests to an LSDB curator for a private opinion
11. Limit links to other LSDBs
12. Consider carefully the transfer of publicly available data from LSDBs to genome browsers

## Background: Develop a Common Ethical Framework

The goal of all such databases is the sharing of genomic and phenotypic information for the benefit of humanity. This requires the protection of privacy, which in this context is the right of the individual and members of their family to be protected against intrusion into their personal information and further intrusions ensuing from access to this, by publication of information. The balance between the public's interest in the value of the shared information, and its interest in the strict protection of privacy has been widely discussed. (For example, by the Academy of Medical Sciences UK in 2006 [http://www.acmedsci.ac.uk/download.php?file=/images/project/Personal.pdf]; adverse comment in [Matthews, 2007]; UK government report in 2009 on genomic medicine [http://www.publications.parliament.uk/pa/ld200809/ldselect/ldsctech/107/10702.htm]; in a commentary from an Islamic perspective [Al Aqeel, 2007]; from the United States [Taylor, 2008]; and in 2003 from the French National Bioethics advisory Committee [http://www.ccne-ethique.fr/docs/en/avis076.pdf]).

This balance will be viewed differently in different cultures [Al Aqeel, 2007] and so international input into detailed guidelines is essential to ensure collective agreement that is requisite to effective collaboration. Harmonization of standards will be a challenge. Although the development of a common ethical framework must be nurtured by culture and country-specific input, the converse also holds true: the guidelines will serve as reference for the developers of national laws and local ethics committees.

For many of the genes and for most of the issues dealt with below it seems likely that an independent group of well-informed individuals to oversee specific LSDBs not only at their initiation but on an ongoing basis will be essential. This general need is underlined by the 2008 revision of the Declaration of Helsinki, http://www.wma.net/en/30publications/10policies/b3/17c.pdf, which states that monitoring of ongoing studies must be put in place in addition to the initial approval by an ethics committee. Governance is thus necessary as new issues may appear in the course of a project or activity.

Inevitably in these guidelines there is a strong emphasis on the validity and complexities of consent and the increasing difficulty of guaranteeing privacy in an era of electronic publishing and growing internet use. The authors would not wish to discourage the curation of LSDBs on this account. Current experience of curators indicates that the majority of patients and research participants are likely to be happy to share their data, although inevitably there will be exceptions, and participants' preference may change based on new understanding of clinical significance. So far, curators have been as likely to receive complaints about the omission of a personal unique variant from the relevant database as its unexpected inclusion.

## Guidelines

The guidelines are presented in approximately the order in which the issues are encountered by the prospective curator:

### Clarify the Main Purpose of the Particular Database, Recognizing That This may Change Over Time

Who does the curator expect will use this LSDB and why?

This will allow evaluation of the exact information required or desirable and whether compliance with the remaining guidelines will be possible with a database open to the public. It may be necessary to decide that at least part of the information should be restricted to identified persons. For discussion of robust methods for validation of identities of enquirers see the GEN2PHEN Knowledge Centre, http://www.gen2phen.org/researcher-identification-primer.

Many LSDBs are used as a tool by diagnostic laboratories assessing the likelihood that the DNA change that they have found is the necessary and sufficient cause of a serious disease and can be used to inform treatment and/or prevention, including preimplantation and prenatal diagnosis and neonatal screening. However, the data needed will vary in different diseases so even for this use the ethical issues will be slightly different for each database. Some examples of questions that must be considered by curators at this stage are shown in Table 2. The answers will allow the generation of a list of ethical requirements that any submitter must fulfil. They will inform decisions about any need for control of access and may also help in the determination of appropriate members of an ethical oversight committee as described later.

**Table 2:** Questions About Aims and Data Required

How much detailed clinical data will be needed and will this be in the form of a link to another database?
Is any family information needed, for example, to support conclusions on pathogenicity?
Will an attempt be made to record every apparently unrelated case with the same mutation?
What ethnic and geographic origin data will be needed and for what purpose?
Will an attempt be made to record all known “neutral” (“normal”) variation?
Is the aim to evaluate the contribution of common variants to common diseases?
Is the goal to inform basic research into the mechanism of disease, for example, modifier genes?
Is the aim to evaluate genetic variation in response to therapy in individuals or populations?
Will the LSDB collect results of in vitro functional analyses?
Will the data include results from a cell or tissue culture of patient/participant material?
Will the database be used to assemble volunteers for new therapies such as mutation-specific strategies?
Is the interest mainly from an evolutionary perspective?

Both those LSDBs which catalog very rare or even unique changes relating to serious disease and those that deal with common variants of small individual effect should adhere to stringent rules of data standardization, validation, quantification, and transparency of sources, as described by participants at the HVP planning meeting [Kaput et al., 2009]. These aims should be clarified and explained in terms understandable by non specialists on the public part of the LSDB Website.

### Define Database Policy With Respect to Sources of Data

This will be dealt with in two main sections: existing data and future data, and within each there is a section on data collected for research and that collected in a diagnostic setting. At the end of this section we suggest a possible consent form that we propose might be appropriate in future, particularly for results obtained in the course of clinical testing.

#### Existing data

##### Published data

Usually, information that is already published and available electronically is assumed to have been collected by the appropriate standards that existed at the time of collection. By virtue of their public availability, these data are generally assumed to be usable in an LSDB. Although it is possible that the person giving the consent for the research and publication may not have foreseen the full implications of Web-based sharing of information, in most situations the likelihood of reidentification and/or misuse of data is considered low. Whenever feasible, the LSDB curator is encouraged to inform the original data producer (defined as corresponding author for the publication) to explore whether any restrictions or modifications of data are appropriate. This recommendation is more for respect of free will and autonomy of patients than for fear of the risk of reidentification. The specifics of a particular rare disease may warrant more stringent monitoring of data; this should be addressed by the ethics oversight committee of the specific LSDB. The main obligation on the curator is to check the scientific accuracy as far as possible, including writing to authors when necessary. Curators should keep in mind that integration of published data may on occasion give rise to conclusions with serious implications for individuals or groups. Similar concerns have been discussed previously, for example, relating to accumulated data on CGH microarrays [Tabor and Cho, 2007]. Occasionally discussion with the oversight group might be needed before full public release of the integrated data.

##### Data existing in diagnostic laboratories or as clinical reports

Available but unpublished diagnostic data are a major problem. This is both because of limited clinical data but also because current practice does not usually ensure that those consenting to genetic testing have given permission for sharing (including scientific publication) of these data beyond the laboratory and clinical team undertaking the analysis. LSDBs must be cautious in accepting unpublished data from any investigators or from accredited diagnostic laboratories, and consider issues that could limit the clinical accuracy of unpublished submissions, including standardization of clinical language, source of data, individual identification, and consent.

It is suggested that where the quality of the data appears to be high and of significant clinical value but it is not feasible to obtain explicit consent, the decisions about which data should be uploaded and also which should be publicly displayed, protected by controlled access or displayed only in summary form should be made by the independent LSDB ethical oversight committee (see point 5). This committee must also be sensitive to different cultural views. In many cases it may be appropriate for these data to be anonymized, that is, made “not identifiable” (see point 6 for explanation of ICH sample coding terminology). Note that the current version of LSDB software LOVD [Fokkema et al., 2005] has the option to store data that are not public but that can be queried. The result of a query hitting nonpublic data is a notification that there is such information in the database but that the curator needs to be contacted to get more information.

In some cases patients/families already report the data themselves (often with a copy of the lab result they obtained), and this can be encouraged with appropriate further information requested if needed. LSDBs should then have a consent form that should be signed by the self-submitter and by all relatives whose results the submitter forwards to the LSDB.

#### Future data

##### Research data

In future research projects, consent forms should specifically indicate what data will be included in a publicly available database and describe their possible intended uses. The clinical significance of published data will continue to change as new findings emerge and the ethical repercussions will depend on many different variables. It may be appropriate to include agreement in the original consent about the need for recontact or for delegation of decision making to an ethical committee for future unforeseen uses and implications.

##### Diagnostic data

With regard to consent, we would strongly recommend that informing donors of the possibility of transmission of data to an LSDB should in the future be part of the consent form for all genetic testing, together with an explanation of why this is useful, and how their privacy will be secured. Refusal to allow inclusion of data in an LSDB should not affect genetic testing because this would contravene the traditional commitment of medicine as exemplified in the UK General Medical Council guidelines 2006: “Make the care of your patient your first concern” (http://www.gmc-uk.org/Good_Medical_Practice_0510.pdf_32611016.pdf).

This should be made clear to the person being asked for consent. It is essential that this be done without coercion in order to preserve the freedom of the consent. However, the information provided to the patients and families should clearly explain the value of gathering such data and mention that in the long term, if data cannot be collected, interpretation of testing results may be less reliable or even impossible, and development of future possibilities for treatment might be compromised. Although the curator should require a statement that the submitter has obtained appropriate consent in whatever way is acceptable in the country of origin of the data, the primary responsibility is that of the submitter. The curator should supply to the testing laboratory a clear explanation about the LSDB on an information sheet that the laboratory can provide to clinicians and patients.

A suggested form of wording as an addition to the consent for diagnostic testing (and which may also be appropriate for testing as part of a research project) is as follows:

1. I understand that the interpretation of DNA test results, including my own is based mainly on publicly available data from others who have been tested before me.
2. I agree that the results of my DNA test and clinical examination may be added to these public data sets, in a manner that does not disclose my personal identity and that is in agreement with data protection law in my country.
3. This information will then be available to help the diagnosis of others, and to further understanding about the disease. Improved understanding of the molecular mechanisms of disease may be important in developing new treatments and/or prevention.
4. Any information that could identify me or members of my family may only be stored when a high standard of privacy and confidentiality (as defined and in accordance with national standards for health data) is maintained. However, unintentional third-party crossexamination of stored nonidentified data might indicate, but not prove, identity. Should this happen, third-party users of the database will undertake not to explore this information further or to contact me.
5. I understand that I will not receive any payment for this.

This wording will need modification in certain circumstances and if any part of the interpretation of the result to be shown on the database depends on family history or testing of other members of the family, this should be considered and consent sought if appropriate.

### Take Specific Communities/Cultures into Account

Identifiable groups such as Ashkenazi Jews or Roma (Gypsies) may be particularly affected by a specific disease and thence become a major part of the relevant LSDB. Following consultation with the community, every effort must be made to take this into account and to provide privacy protection and respect cultural sensitivity ensuring that high ethical standards are maintained. A small specialized database gives the greatest chance of the unintended identifiability of one of the subjects. It may occasionally be necessary to store data only at summary level to preserve anonymity, as has been done in the Israeli and other National/Ethnic Mutation Databases (NEMDBs) [Patrinos, 2006; Zlotogora et al., 2007]. The cultural sensitivity of particular groups such as the Maori of New Zealand will need a step of local consultation before any sharing of DNA data, even for disorders not especially prevalent in that group.

### Take Vulnerable Persons into Account

Persons who do not have the capacity to consent either because of disability or young age are especially vulnerable. In some disorders, this will apply to many of the patients/ participants and regular external review of procedures for obtaining consent from appropriate relatives/representatives or other suitable authority should be in place. Usually this will be part of the remit of the LSDB oversight committee.

### Create an Ethics Oversight Committee

A variation database that accepts genotype and phenotype data not already in the public domain (or that are in the public domain but whose combination and integration are foreseen to change the degree of identifiability of persons) and makes them widely available, should have an independent and well-informed oversight group. This should be drawn from several disciplines and from relevant society stakeholders, including patient groups, to review the particular ethical issues arising in relation to that LSDB. They should recommend constraints needed in uploading and displaying data and decide on any requirement for control of access or for anonymization. The delegation of such decisions to a committee with a long-term remit will balance the difficulty of having truly informed consent in such a fast moving field. The decisions of this committee may require formal ethical approval either from their own institution or a national body.

A database that only accepts publicly available data would still benefit from some independent ethical review, and this is strongly recommended This could be provided either by a specific oversight committee as described above, or perhaps by an international HGVS or HVP group who could advise a number of databases.

### Remove Identifying Information Before Submission to Database

Every effort must be made to ensure that the individuals whose DNA variation is displayed in an LSDB are not individually identifiable. With increasing availability of total genomic sequence and the enormous amount of personal information retrievable from websites, absolute certainty of nonidentifiability is no longer guaranteed [Barash, 2007; Homer et al., 2008; Lowrance and Collins, 2007; Walter, 2007]. However, with care, the risk of identification from an LSDB will be very low in almost every case, particularly if data from genome-wide analyses such as single nucleotide polymorphism (SNP) genotyping data are not associated with the mutation (see point 7 for possible exceptions such as unique variants). There is now a set of definitions including sample coding terminology agreed internationally and recognized by all constituents of the International Conference on Harmonisation (ICH) that has become official in 2008 (http://www.fda.gov/downloads/RegulatoryInformation/Guidances/ucm129296.pdf).

These definitions will be used here and are explained below.

Decide whether data should be “coded” (also called “reidentifiable”) or “anonymized” (also called “deidentified”). For the purposes of these guidelines, “coded” is taken to mean removal of all existing identifiers as far as is compatible with usefulness of the data and substitution by proxy identifiers that are used in the database. The link between the existing identifier and the proxy identifier could be maintained either by the submitter acting as the “honest broker” between the hospital records and the LSDB or within a securely nonpublic area of the LSDB. It would be desirable that each proxy identifier be unique and generated by a standard coding mechanism, perhaps by some national or international body. This would avoid inadvertent duplication of identifiers that might arise if the process was left to individual LSDB curators. For published data, and especially for recent publications, many cases are already coded and then classified as “reidentifiable” specifically for publication. These codes might be acceptable if not recorded in hospital notes. However, new coding for the database would be safer. Unpublished data, unless anonymized as explained below, should always be recoded to make them reidentifiable and not directly identifiable. In the case of unpublished data on a rare disorder it is advisable that no link between a particular entry and the submitter should be displayed, and that ethnic origin and geographic data on the donor are not visible. This is recommended even if the clinical data displayed are minimal.

Anonymized (or “deidentified”) here means that identifiers and any information that might be used as clues to identity through other links are permanently removed and the link to the ID used by the submitter is destroyed. This would limit the usefulness of the data, particularly with regard to long-term phenotype follow-up data and any late correction of wrong information but also possibly in ways that cannot currently be predicted. It also makes withdrawal of consent impossible, and is not the approach of choice.

If the decision is to proceed with coded or in other words “reidentifiable” data, although the original identifier is replaced by a code, many other pieces of information give possible clues to identity and will need to be removed to avoid unauthorised reidentification and, in many countries, to obey privacy laws. In most published cases, all data given in the publication would be acceptable to include in the database, including geographical location and ethnic group of individuals studied (both for cases and for population controls) and clinical details of patients. Limited family details present in the original report that help in the interpretation of the mutation may also be included. A link to the original publication is acceptable and useful. Note that data on frequency and population/ethnicity can be very helpful in the design of cost-effective targeted diagnostics and/or treatment protocols. For unpublished data, the specific nature of the LSDB will be considered by the ethical oversight committee in recommending which data can be collected and displayed. Special consideration should be given to rare mutations (see point 7).

### Add Further Protection of Confidentiality if Needed

This may be necessary in the case of rare or unique mutations in rare diseases, unique combinations of clinical features, or where higher protection is required for some other reason. The oversight committee will be valuable here. The database will have limited usefulness if important genotype–phenotype data cannot be released because this information alone might allow identification of the individual. In diseases where very detailed clinical data have been collected (especially clinical photographs or detailed pedigrees) access to these data may have to be restricted by appropriate registration and approval for access. A possible solution here that could also be applied to any unpublished data for which explicit consent is not certain would be to display the mutation in the database with no other data at all. Someone with a genuine reason for wanting to know if this mutation causes disease could then click on a tool that would send an e-mail to the curator to explore the possibility of finding more information (see Section 8). There are several variations to this approach and the display of every variant recorded, including those for which no other information can be displayed publicly, makes accurate enquiries easier for the user rather than requiring the enquirer to specify, in correct format, a variant which is not displayed. As mentioned in point 2, one of us (J.dD.) has already provided a similar facility for LOVD databases [Fokkema et al., 2005].

### Allow no Further Disclosure Without Consent

Information about a particular entry beyond what is publicly viewable in the database should not be supplied by a curator to an enquirer unless consent for this has been explicitly provided. The request should be referred to the submitter who will use using professional judgement in their response. This will usually require seeking further explicit consent from the patient. If there is any doubt the independent oversight committee should be consulted.

### Make Provision for Removal of Data from Database

The parents or guardians who have given consent for a child's or incompetent adult's information to be included in the LSDB should be made aware of their right to withdraw this information at any time (unless data are truly not identifiable). The LSDB should make available information in order to facilitate this task. If a child reaches the age of consent (16 in many but not all countries) and is capable of making a decision, those who previously authorized data sharing should ensure that he/she is aware of his/her LSDB entry and has the right to withdraw it. This should also be the case if an adult previously judged incompetent becomes competent. However, it should be made clear that although it will be possible to eradicate information that was originally displayed from the database, it may not be possible to eradicate it from other sources that have used this information, for example, in an overview publication.

### Be Cautious in Response to Requests to an LSDB Curator for a Private Opinion

On whether a particular variant is pathogenic, especially if any of the information used is not published. Add a disclaimer about responsibility for the clinical use of the opinion and be cautious of a “virtual medical advisor relationship.” From the medicolegal point of view, it is safest to obtain clinical interpretation from published data. If unpublished information is used, a careful record should be kept. Recommendations of the IARC Working Group on Unclassified Genetic Variants encourage that classification of pathogenicity be carried out not by individuals but by teams of experts that can carefully evaluate all lines of evidence [Greenblatt et al., 2008; Tavtigian et al., 2008].

### Limit Links to Other LSDBs

If mutations in more than one gene are relevant to a particular disease, it may be useful to record the variation of both genes in the same individual and link the entries so that the fact that it is one person is recorded. This facility is already available on at least one LSDB platform (LOVD) and can be of great value in the interpretation of results. However, logically it may eventually extend to enough genes to allow identification of the individual. At this point, the considerations of the ethics of large scale resequencing will be relevant (see [Lowrance and Collins, 2007]). For example, a recent investigation into DNA variants causing X-linked mental retardation included substantial amounts of sequence information on the coding regions of X-linked genes [Tarpey et al., 2009]. These data were regarded as too sensitive for the full set of variants for each patient to be entered onto the LSDBs in the most informative way. See http://www.LOVD.nl/MRfor the summary data submitted.

### Consider Carefully the Transfer of Publicly Available Data from LSDBs to Genome Browsers

Some of the genetic variations collected and displayed by curators of LSDBs are now visible also in one or several of the main tools used by scientists worldwide analyzing the human genome, for example, at the National Center for Bioinformatic Information (NCBI) at Bethesda, and the UCSC Genome Browser at Santa Cruz. This does not raise entirely new ethical issues except in the need for adequate recognition of the work of the LSDB curator. It makes the misuse of data for reidentification slightly more likely and may also increase the chance of a mistake being widely disseminated in a short time. Further discussion of the sharing of data with genome browsers can be found elsewhere [den Dunnen et al., 2009].
